# Electrodiagnostic Examination of the Tibial Nerve in Clinically Normal Ferrets

**DOI:** 10.4061/2010/756321

**Published:** 2010-07-27

**Authors:** Ezio Bianchi, Daniela Callegari, Manuela Ravera, Maurizio Dondi

**Affiliations:** Animal Health Department, University of Parma, Via del Taglio 10, 43126 Parma, Italy

## Abstract

Tibial nerves of 10 normal domestic ferrets (*Mustela putorius furo*) were evaluated by means of electrodiagnostic tests: motor nerve conduction studies (MNCSs), supramaximal repetitive nerve stimulation (SRNS), *F* waves, and cord dorsum potentials (CDPs). Values of conduction velocity, proximal and distal compound muscular action potentials, and amplitudes of MNCS were, respectively, 63.25 ± 7.56 m/sec, 10.79 ± 2.75 mV, and 13.02 ± 3.41 mV. Mean decrements in amplitude and area of compound muscular action potentials of wave 9 with low frequency SRNS were 0.3 ± 3.83% and 0.1 ± 3.51%. The minimum latency of the *F* waves and the *F* ratio were, respectively, 8.49 ± 0.65 ms and 1.92 ± 0.17. Onset latency of CDP was 1.99 ± 0.03 ms. These tests may help in diagnosing neuromuscular disorders and in better characterizing the hindlimb paresis reported in many ferrets with systemic illnesses.

## 1. Introduction

Clinical signs of neurological disorders are frequently reported in the domestic ferret (*Mustela putorius furo*). The most common presenting complaint is paraparesis/paralysis and ataxia [1]. These clinical presentations in a ferret are caused by primary neurological diseases or, more often, by systemic illnesses [2, 3]. Indeed, systemic disorders like endocrine and metabolic problems may cause secondary involvement of the nervous system. However, any systemic disease may cause weakness that mimics neurological involvement [1]. An accurate neurological examination is always mandatory in these cases in order to try to identify the presence of a neurological problem and to localize it. Electrodiagnostic tests represent an extension of the neurological examination useful, especially in neuromuscular problems, in precisely localizing the disorder and in defining the nature of the lesion. Electrodiagnostic procedures, as the other neurodiagnostic tests, have only been marginally investigated in pet ferrets [1, 2, 4]. In particular, most of the reference values for electrodiagnostic tests have still not been determined in clinically normal ferrets. The objective of the present study was to describe for the first time the electrophysiological evaluation of the tibial nerve in this species using the following electrodiagnostic tests: motor nerve conduction studies (MNCS), supramaximal repetitive nerve stimulation (SRNS), *F* waves, and cord dorsum potentials (CDP). A further objective was to establish the reference values for the main parameters of each of these tests in domestic ferrets.

## 2. Materials and Methods

### 2.1. Animals

The tests were performed on 10 client-owned domestic ferrets (6 females and 4 males) of different colours, from 8 months to 2 years of age, with an average weight of 1.07 kilograms, presented for evaluation of auditory function. Brainstem auditory evoked potentials (BAEP) testing was requested in these subjects in the frame of breeding programs aimed at reducing the incidence of congenital deafness. None of the subjects had a history of neurologic problems and physical and neurological examinations were unremarkable. Informed consent of clients was obtained prior to participation of ferrets in this study.

### 2.2. Procedure

**Figure 1:**
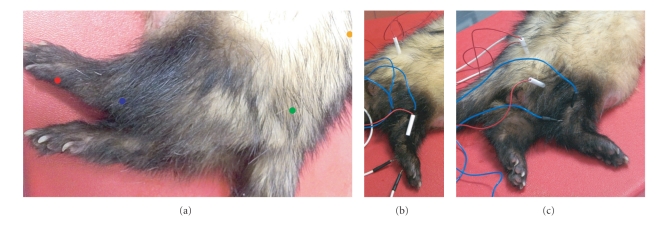
(a) Schematic representation of the points of stimulation and recording of the electrodiagnostic tests. Red dot: plantar interosseous muscles. Blue dot: stimulation of the sciatic-tibial nerve at the tarsus. Green dot: stimulation of the sciatic-tibial nerve at the femoral neck. Orange dot: recording of the CDP at the intervertebral space L4-L5. (b) Image of MNCS of the right sciatic-tibial nerve showing the points of insertion of the needle electrodes used for proximal stimulation (red and white wires) distal stimulation (blue and black wires), and recording (white and black wires). The single red and white wire whose needle is inserted between distal stimulation and recording is the ground electrode. (c) Image of the CDP test of the right tibial nerve showing the points of insertion of the needle electrodes used for stimulation (blue and black wires) and recording (white and black wires). The red and white wires whose needles are inserted between stimulation and recording are the ground electrode and the electrodes used for the proximal stimulation during MNCS.

All ferrets in the study were sedated with an intramuscular injection of 80 mcg/kg of medetomidine (Domitor; Pfizer). The procedures were performed, before BAEP tests, with the ferrets in lateral recumbency while rectal temperatures were monitored and maintained above 36.5°C by using heating pads. At the end of the tests, sedation was reversed with an intramuscular injection of 400 mcg/kg of atipamezole (Antisedan; Pfizer). Stimulation: The sciatic-tibial nerve was stimulated at the level of the femoral neck (MNCS) and at the tarsus (MNCS, SRNS, *F* waves, CDP) (Figures 1(a) and 1(b)). Needle electrodes were used; the cathode was located approximately 1 cm distally to the anode at the femoral neck and at the tarsus for MNCS and SRNS. Four *F* waves and CDP cathode and anode were inverted. The electrical stimulus applied was a square wave of supramaximal intensity, 0.1 milliseconds of duration, and a frequency of 1 Hz for MNCS and *F* waves, 2 Hz for SRNS, and 5 Hz for CDP. Recording: The compound muscular action potentials (CMAP) were recorded from the plantar interosseous muscles with the recording needle inserted in the muscle, and the reference needle placed distally in the subcutis of the plantar surface of the hind paw (Figures 1(a) and 1(b)). Trains of 9 stimuli were delivered to the nerve for SRNS. The *F* waves elicited by at least 20 stimuli were recorded. The *F* ratio was calculated in each subject by the formula: *F* ratio = (*F* − *M* − 1)/2*M*, where *F* represents the latency of the *F* wave and *M* that of the CMAP (also known as *M* response). This ratio provides a comparative assessment of motor conduction between proximal and distal nerve segments. For CDP, the recording needle electrode was inserted in the subcutis at the intervertebral space L4-L5 and the reference electrode placed subcutaneously 2-3 cm laterally on the controlateral side of the spine (Figures 1(a) and 1(c)). Two series of 250–500 stimulations were averaged for each subject to verify repeatability of the potentials recorded. The ground needle electrode was inserted in the subcutis between stimulation and recording (Figures 1(b) and 1(c)). The tests were performed in all the ferrets using the same electromyographic equipment (Myoquick, Micromed, Mogliano Veneto (TV)—Italy). Filters were set at 20–2000 Hz for SRNS and *F* waves, 20–5000 Hz for MNCS and 30–2500 Hz for CDP. In each subject only one tibial nerve was evaluated and the total duration of the procedures ranged between 10 and 15 minutes. Descriptive statistics were calculated for the main parameters of each test.

## 3. Results

**Figure 2:**
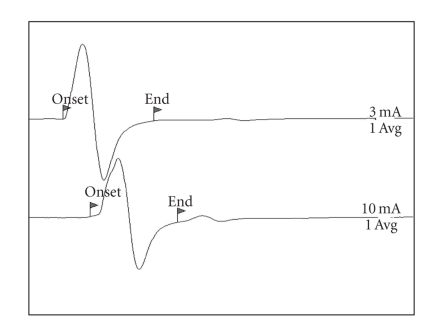
MNCS of the left sciatic-tibial nerve on ferret N.2. 2 ms/Div; 5 mV/Div.

The CMAP waveform was characterized by a biphasic shape with initial negativity (Figure 2). Conduction velocity, proximal, and distal CMAP amplitudes of MNCS of sciatic-tibial nerve were respectively 63.25 ± 7.56 m/sec, 10.79 ± 2.75 mV, and 13.02 ± 3.41 mV (Table 1).

**Figure 3:**
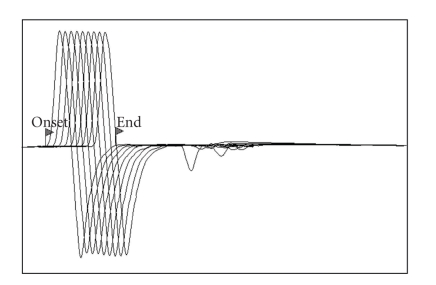
SRNS of the right tibial nerve of ferret N.5. 2 ms/Div; 2 mV/Div. Stimulation rate of 2 Hz.

Mean decrements in amplitude and area of CMAP of wave 9 during SRNS were respectively 0.3 ± 3.83% and 0.1 ± 3.51% (Figure 3). 

**Figure 4:**
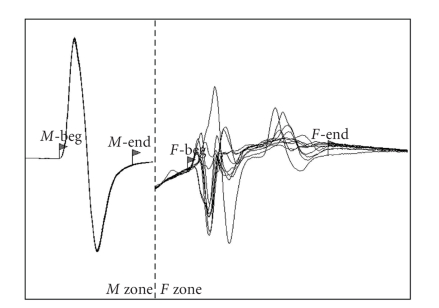
*F* waves of the right tibial nerve of ferret N.8. 2 ms/Div; 2 mV/Div (*M* Zone); 0.2 mV/Div (*F* Zone).

The *F* waves were polyphasic had a minimum latency of 8.49 ± 0.65 ms, maximum amplitudes of 942.9 ± 647.56 *μ*V and an *F* ratio of 1.92 ± 0.17 (Figure 4). *F* waves frequency was 100% in 8 subjects, 90% and 40% in the remaining 2 ferrets (Table 2). 

**Figure 5:**
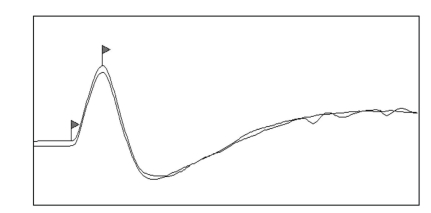
CDP of the left tibial nerve of ferret N.6. Left flag indicates the onset; right flag indicates the peak of the potential. 2 ms/Div; 5 *μ*V/Div.

CDP consisted of a large negative peak followed by a long-latency positive deflection (Figure 5). CDP onset latency was 1.99 ± 0.03 ms, onset-to-peak latency difference was 1.85 ± 0.36 ms and peak amplitude was 4.92 ± 2.93 *μ*V (Table 3).

## 4. Discussion

The tibial nerve is a long mixed nerve and represents the more caudal terminal branch of the sciatic nerve. In the present study, this nerve was chosen because its investigation may help in clarifying the nature of hindlimb paresis often reported in this species. Further, it has the advantage over the other nerves of the posterior limb that the distance between the points of stimulation of MNCS is longer and this improves the accuracy of determination of conduction velocity [5]. The stimulation and recording sites of this nerve were similar to those commonly used in dogs and cats, as were the waveforms recorded (Figures 1–5). MNCS was the first test performed to precisely identify the optimal stimulation sites. This test studies the motor fibres of the nerve and may help to identify the nature of the lesion (mainly demyelinating versus mainly axonal features). In neuropathies, a generalized reduction in CMAP amplitudes is usually indicative of axonopathy, while a slowed conduction velocity and a prolonged duration of CMAP are features of demyelination. At the authors' knowledge, this is the only electrodiagnostic test for which some normal values were recently reported [4]. The reference values provided in this case report (CMAP amplitudes and conduction velocity) were slightly less than those founded in our subjects. The reduced amplitudes were probably dependent from the use of surface cutaneous electrodes for recording instead of the monopolar needles used in our study. It can be hypothesized that other factors not described in detail in this clinical case, like the methods of stimulation and the composition of the control group, were responsible for the difference with our normal values of conduction velocity. 

SRNS is a test that evaluates neuromuscular transmission and is therefore useful in the diagnosis of junctionopathies. It was performed after MNCS and showed decrements similar to those reported in normal dogs and cats using the same frequency of stimulation (Figure 3) [6, 7]. None of the ferrets evaluated had decrements of the CMAP of more than 8% during the stimulation train. In a recent report of a case of myasthenia gravis in a ferret by the 3rd response, a decrement of 45.5% was found [4]. 

The potentials recorded during *F* waves studies could be evoked only with supramaximal stimuli; therefore, they were probably constituted mainly by *F* waves. In fact, the supramaximal stimulation of a motor nerve produces the orthodromic “centrifugal” nerve impulse responsible for the first muscle contraction (*M* response) and antidromic “centripetal” motor nerve activation. When this nerve impulse reaches the cell body, it gives rise to a new orthodromically conducted signal that produces a new smaller muscular contraction (*F* waves) several milliseconds after the *M* wave. A contamination of *F* waves by the long-latency muscle action potentials produced by the electrically elicited stretch reflex (*H* reflex) cannot be excluded. *F* waves represent purely motor events and reflect the integrity of the entire motor fibre from the motor neuron to the terminal branch [8]. This test, like MNCS, is affected in motor neuropathies and is especially useful in detecting problems of the proximal and distal segments of the motor fibres of the nerves (polyradiculoneuritis, distal denervating disease, etc.).

CDP are spinal cord field potentials produced by the propagation of sensory action potentials into the spinal cord. They represent purely sensitive events as they reflect the function of sensory fibres of spinal nerves [6]. These potentials consisted of a large negative peak followed by a long-latency positive deflection (Figure 5). CDP are affected in sensory neuropathies, radiculopathies involving dorsal roots, and in disorders affecting spinal cord dorsal horns. The onset latency of CDP and the minimum latency of *F* waves were stable among subjects as expected for the lack of limb length variability of this species. On the contrary, similarly to what reported in other animals and persons, the amplitudes of CMAP of MNCS, *F* waves, and CDP potentials where somehow more variable [5, 6, 8].

## 5. Conclusions

To the best of our knowledge, this is the first report that extensively describes methodologies of tibial nerve electrodiagnostic investigation, including more advanced tests like SRNS, *F* waves, and CDP, and provides normal values of neuromuscular electrodiagnostic studies in domestic ferrets. These tests, combined with electromyography, may add useful diagnostic information in neuromuscular diseases of ferrets like Botulism, Toxoplasmosis, endocrine and paraneoplastic neuropathies, myasthenia gravis, disseminated idiopathic myositis, and so forth. [1, 2, 4, 9, 10]. The availability of reference values is especially important for symmetrical disorders, like hindlimb paresis, in which the contralateral limb of the same patient cannot be used as a normal control. Moreover, electrodiagnostic tests, together with a complete neurologic examination and with other collateral diagnostic procedures like muscle and nerve biopsies may help in better understanding and characterizing the “hindlimb weakness” reported in many subjects with systemic illnesses. 

More studies including subjects of different ages and other electrodiagnostic tests and nerves are needed to further increase the information available for investigation of disorders affecting the peripheral nervous system of ferrets.

## Figures and Tables

**Table 1:** Values of motor nerve conduction studies (MNCS) of sciatic-tibial nerve.

Ferret	Proximal latency (ms)	Proximal duration (ms)	Proximal amplitude (mV)	Proximal area (mV/ms)	Distal latency (ms)	Distal duration (ms)	Distal amplitude (mV)	Distal area (mV/ms)	Conduction velocity (m/sec)
N.1	2.8	2.2	7.1	3.7	1.4	2.3	17.4	8.9	53.6
N.2	3.2	4.9	16.8	18	1.8	4.7	20.6	20	64.3
N.3	3	4.8	9.4	7.9	0.9	4.7	10.5	6.3	60
N.4	2.9	4.7	11	9.6	1.6	4.6	13.3	11	69.2
N.5	2.4	4.1	7.6	6.1	1.2	3.8	9.9	7.1	66.7
N.6	2.4	4.1	11.1	10	1.5	3.7	12.8	10	77.8
N.7	2.8	4.5	12	11	1.6	4.2	12.2	11	62.5
N.8	2.9	4.2	11.5	9.6	1.6	4.3	11.2	8.9	67.7
N.9	2.7	5	12.1	11	1.3	4.8	11.7	9.7	57.1
N.10	2.6	3.4	9.3	7.4	1.2	3.5	10.6	7.9	53.6
**MEAN**	**2.77**	**4.19**	**10.79**	**9.43**	**1.41**	**4.06**	**13.02**	**10.08**	**63.25**
**SD**	0.25	0.85	2.75	3.79	0.26	0.77	3.41	3.81	7.56

**Table 2:** Values of *F* waves of tibial nerve stimulated at the tarsus.

Ferret	Minimum latency (ms)	*F* ratio	Maximum amplitude (*μ*V)	Frequency (%)
N.1	8.5	2.1	308	40
N.2	9	2	417	90
N.3	8.9	2	642	100
N.4	9.2	2.1	751	100
N.5	7.8	2	1600	100
N.6	7.3	1.7	544	100
N.7	8	1.6	472	100
N.8	8.7	1.8	895	100
N.9	9.3	1.9	2300	100
N.10	8.2	2	1500	100
**MEAN**	**8.49**	**1.92**	**942.9**	
**SD**	0.65	0.17	647.56	

**Table 3:** Values of cord dorsum potential (CDP) of tibial nerve stimulated at the tarsus and recorded at L4-L5.

Ferret	Onset latency (ms)	Onset/Peak latency (ms)	Amplitude (*μ*V)
N.1	2.01	2.08	5.94
N.2	2.01	1.41	3.04
N.3	1.95	1.83	2.8
N.4	1.95	2.14	1.94
N.5	2.01	1.59	4.5
N.6	1.95	1.59	7.77
N.7	2.01	2.08	3.56
N.8	1.95	1.28	7.52
N.9	2.01	2.14	1.63
N.10	2.01	2.32	10.53
**MEAN**	**1.99**	**1.85**	**4.92**
**SD**	0.03	0.36	2.93
